# The Social Roots of Suicide: Theorizing How the External Social World Matters to Suicide and Suicide Prevention

**DOI:** 10.3389/fpsyg.2021.621569

**Published:** 2021-03-31

**Authors:** Anna S. Mueller, Seth Abrutyn, Bernice Pescosolido, Sarah Diefendorf

**Affiliations:** ^1^Department of Sociology, Indiana University Bloomington, Bloomington, IN, United States; ^2^Department of Sociology, The University of British Columbia, Vancouver, BC, Canada; ^3^Department of Political Science, The University of Utah, Salt Lake City, UT, United States

**Keywords:** Sociology, Durkheim, suicide, social connectedness, social networks, social psychology, suicide contagion, suicide prevention

## Abstract

The past 20 years have seen dramatic rises in suicide rates in the United States and other countries around the world. These trends have been identified as a public health crisis in urgent need of new solutions and have spurred significant research efforts to improve our understanding of suicide and strategies to prevent it. Unfortunately, despite making significant contributions to the founding of suicidology – through Emile Durkheim’s classic Suicide (1897/1951) – sociology’s role has been less prominent in contemporary efforts to address these tragic trends, though as we will show, sociological theories offer great promise for advancing our understanding of suicide and improving the efficacy of suicide prevention. Here, we review sociological theory and empirical research on suicide. We begin where all sociologists must: with Durkheim. However, we offer a more comprehensive understanding of Durkheim’s insights into suicide than the prior reviews provided by those in other disciplines. In so doing, we reveal the nuance and richness of Durkheim’s insights that have been largely lost in modern suicidology, despite being foundational to all sociological theories of suicide – even those that have moved beyond his model. We proceed to discuss broadly acknowledged limitations to Durkheim’s theory of suicide and review how more recent theoretical efforts have not only addressed those concerns, but have done so by bringing a larger swatch of sociology’s theoretical and empirical toolkit to bare on suicide. Specifically, we review how recent sociological theories of suicide have incorporated insights from social network theories, cultural sociology, sociology of emotions, and sociological social psychology to better theorize how the external social world matters to individual psychological pain and suffering. We conclude by making explicit bridges between sociological and psychological theories of suicide; by noting important limitations in knowledge about suicide – particularly regarding the roles of organizations, inequality, and intersectionality in suicide – that sociology is well situated to help address.

## Introduction

The past 20 years have seen dramatic rises in suicide rates in the United States and other countries around the world (Curtin et al., 2016; Lee et al., 2018; Martini et al., 2019). These trends have been identified as a public health crisis in urgent need of new solutions (Office of the Surgeon General and National Action Alliance for Suicide Prevention, 2012) and have spurred significant research efforts to improve our understanding of suicide (Joiner, 2005; Klonsky and May, 2015; O’Connor and Kirtley, 2018) and strategies to prevent it (Wyman et al., 2010; Wasserman et al., 2015). Unfortunately, despite making significant contributions to the founding of suicidology – through Emile Durkheim’s (1897/1951) classic *Suicide* – sociology’s role has been less prominent in contemporary efforts to address these tragic trends, though as we will show, sociological theories offer great promise for advancing our understanding of suicide and improving the efficacy of suicide prevention.

Here, we review sociological theories of suicide with the explicit goal of building bridges. We begin where all sociologists must: with Durkheim. However, we offer a more comprehensive understanding of Durkheim’s insights into suicide than prior reviews provided by non-sociologists (Joiner, 2005). This is critical. Much of the nuance and richness of Durkheim’s insights have been lost in modern suicidology, and yet Durkheim is foundational to understanding sociological theories of suicide, as well as understanding the potential of sociology for suicidology. We also discuss limitations in the Durkheimian approach and how more recent efforts have not only addressed those concerns but have done so by bringing sociology’s broader theoretical and empirical toolkit to bare on suicide. These insights draw largely from social network theories, cultural sociology, sociology of emotions, and sociological social psychology. We conclude by making explicit bridges between sociological and psychological theories of suicide and by noting important limitations in knowledge about suicide – particularly regarding the roles of organizations, inequality, and intersectionality – that sociological scholarship is uniquely prepared to address.

### Durkheim Explained

The sociological study of suicide remains rooted in founder Émile Durkheim’s (1897/1951) empirical study of suicide, still the disciplines’ greatest contribution to suicidology (Joiner, 2005). Durkheim’s theory posits two core principles: (1) that the structure of suicide rates is a positive function of the structure of a group or class of people’s social relationships and those (2) that social relationships vary according to their level of integration and (moral) regulation. Though Durkheim never clearly defined his dimensions, sociologists have generally treated integration as the structural elements of social relationships like the number and density of ties (Pescosolido, 1990, 1994; Bearman, 1991) and regulation as the degree to which a collective’s moral order controls and coordinates its member’s attitudes and behaviors (Bjarnason, 1998; Abrutyn and Mueller, 2016). Additionally, Durkheim articulated two continua and four types of suicide related to integration and regulation: egoistic/altruistic suicides (too little ↔ too much *integration*) and anomic/fatalistic suicides (too little ↔ too much *regulation*).

Importantly, Durkheim was not interested in the subjective appraisals suicide decedents provided for why they chose suicide, but rather saw suicide, like alcohol abuse or homicide, as a symptom of collective breakdown of society. In turn, rather than focus interventions to reduce suicide on individuals, he argued [like many population health scientists today (Pescosolido, 1992; Hall and Lamont, 2009)] that a more efficacious avenue to protect individual well-being lies in collective public projects to produce protective structural changes. These changes can restore the integrative and regulative functions of the social groups to which individuals belong or lessen the intense pressure on individuals in social groups where integration and regulation have exceeded “healthy” levels. Durkheim was writing at a time of immense political, economic, and cultural change, which in turn motivated his emphasis on the types of suicide predicated on *too little* integration or regulation over the dangers of *too much*. Consequently, empirical research examining when and why connectedness or moral clarity might prove fatal to a group’s members was sidelined until rather recently; a point we will return to below.

#### Integration and Suicide

Of the two social factors, Durkheim’s *integration* has had the most profound impact on both sociology and suicidology. In explaining the power of integration, Durkheim argued that the more extensive and denser a collective’s social relationships – i.e., the more integrated the collective – the more enmeshed individual group members become, and, therefore, the more meaning and purpose individuals feel about their lives. He remarked, “The bond that unites [individuals] with the [group] attaches them to life [and] prevents their feeling personal troubles so deeply (1951:209–210).” He continues that suffering physically, psychologically, or spiritually, “does not exist for the believer firm in his faith or the man strongly bound by ties of domestic or political society” (ibid., 212). This collective belonging protects individuals from what Durkheim termed “egoistic” suicide, or suicides resulting from isolation and a lack of collective belonging. Integration, then, is borne of the recurring social relationships that require tending and care, and which are embedded in larger networks that form groups, communities, or perhaps, even nation-states. This includes being tied to families and neighborhoods (Bjarnason, 1994; Maimon and Kuhl, 2008; Maimon et al., 2010) as well as communities (Baller and Richardson, 2002). These relationships provide members with what sociologists call social capital, or tangible and intangible benefits built on membership (Coleman, 1988; Portes, 2014).

In recent theories of suicidology, integration has been operationalized through perceptions about belongingness (Joiner, 2005) and connectedness (Centers for Disease Control and Prevention, n.d.; Klonsky and May, 2015). However, Durkheim was not interested in *perceptions* or *appraisals*, which he argued were subjective. Instead, integration is meant to be a characteristic of the group, not of individuals (Turner, 1981; Pescosolido, 1994; Mueller and Abrutyn, 2016). Regardless, Durkheim’s basic premise – that being highly integrated (whether measured at the collective level or through individual perceptions) is protective against suicide – has received consistent strong empirical support across time and space and disciplinary boundaries (Stack, 2000; Joiner, 2005; Wray et al., 2011).

Conversely, the flipside of egoistic suicide – suicides caused by too much integration or *altruistic* suicide – has received scant theoretical and empirical attention (Davies and Neal, 2000; Stack, 2004). In Durkheim’s estimation, tight-knit societies could rob individuals of their ability to make decisions under certain conditions, leading to suicides for the “good of the group.” He pointed, for instance, to Hindu *Sati*, a rare form of suicide in which Hindu widows are compelled to throw themselves on their husband’s funeral pyre (Abrutyn, 2017). Though Durkheim thought over-integrated suicides relics of earlier forms of society, Abrutyn and Mueller (2016) have argued they are more common than we think. Pointing to the literature on social capital (Coleman, 1988; Portes, 2014) and on suicide clusters (Niedzwiedz et al., 2014), they argue that in the meso-level of society, we can find numerous examples of communities where social structure can be exceedingly dense, like some religious communities (Coleman, 1988), high schools and neighborhoods (Mueller and Abrutyn, 2016), army bases, and institutions like prisons or psych wards (Abrutyn and Mueller, 2018). Indeed, many of these places are disproportionately vulnerable to the emergence of suicide clusters (Haw et al., 2013). This highlights potential downsides to connectedness, such as groupthink or high costs for non-conformity (Portes, 2014) and cautions scholars from positing connectedness as a purely protective phenomena.

#### Regulation and Suicide

Durkheim also argued that suicide rates were related to the degree to which a given group’s rules and social norms were consensually clear, coherent, and shared. Living in a poorly regulated society or social group resulted in what Durkheim termed “anomic” suicides. In essence, Durkheim posited that humans, as animals, were not inherently moral creatures, but had to acquire morality from without. Notably, “moral” was synonymous with “social” in Durkheim’s day, and thus he saw social bonds as having integrative features like intimacy *and* regulative features like moral obligations and expectations. Thus, Durkheim set up several routes to de-regulation causing suicide. First, societies where norms were constantly changing and or where there was a general breakdown in moral clarity, people’s ability to easily identify their purpose would be constantly under attack. Second, regulation could suddenly be weakened, either by a change of status in the individual (e.g., losing a job) or by a collective crisis (e.g., an economic recession or global pandemic) that challenged society’s ability to provide clear moral or social guidance. In short, Durkheim saw a sense of shared moral clarity as an independent force providing protection to members of a group. While Durkheim emphasized the societal level, it is important to note that we can also develop moral relationships with a group (Lawler et al., 2009) and an abstract system of norms (Abrutyn and Lizardo, 2020), which expands the “web” in which a given person may find themselves protected.

Like integration, too much regulation may also cause what Durkheim termed “fatalistic” suicide. For Durkheim, fatalistic suicides occurred when members of a group or social category were subjected to intense psychic and physical coercion such that there was no hope for a future without suffering. Though Baumeister (1990) has argued, suicide is very often about escape from pain, like other Durkheimian types, fatalistic suicides refer to a class of suicides that are not limited by specific individual motives. To date, few studies have explicitly explored Durkheim’s fatalistic suicide, though we can provide some examples of its possible research potential. First, structural inequality or violent oppression within families or communities may render groups of oppressed individuals disproportionately vulnerable to (fatalistic) suicides. For example, we know that women in violent relationships often feel trapped and over-regulated (Summers-Effler, 2004); and are more susceptible to suicidality (Chang, 1996). Women in rural China or Iran, for instance (Fei, 2010; also, Aliverdinia and Pridemore, 2009), may also fit this pattern, as may women of color who emigrate to another country and find themselves in precarious employment situations (van Bergen et al., 2009). Second, suicide bombers are often over-regulated (by some military or colonizing political system, as well as over-integrated into their local community), which may produce the type of structure that delimits options for resisting and expressing one’s obligations to their community (Pedahzur et al., 2003; Abdel-Khalek, 2004).

### Durkheim’s Limitations

Despite the importance of Durkheim’s theory to suicidology generally, and sociology of suicide more specifically, Durkheim’s theory is not without limitations which have in turn shaped more contemporary sociological theories of suicide.

One of the oldest and most notable limitations of Durkheim is methodological. Durkheim fails to adequately address the ecological fallacy of studying suicide rates to understand individual behavior. Durkheim forcefully argued that societal- or macro-level forces (integration and regulation) *caused* individual-level behavior (suicide), and yet the link between societal-level social forces and individual behavior is challenging yet crucial to document. Compounding Durkheim’s methodological limitations was the intellectual climate of his day. As a nascent discipline, Durkheim worked hard to distinguish and legitimize sociology apart from psychology and anthropology. Hence, using social psychological or cultural ideas – two sets of phenomena associated, respectively, with the other disciplines – was impossible. He could not, for instance, think about identity or emotions in sociological terms and, therefore, could not bring sociology into the micro-level of social reality. As we shall see, this limitation, as well as Durkheim’s explicit rejection of Gabriel Tarde’s imitation theory (Abrutyn and Mueller, 2014b), has also constrained contemporary sociologists, until rather recently, from thinking about how suicide may spread from one person to another. Finally, Durkheim’s own lack of attention to power and inequality, and the legacy it has generated, represents a major limitation. Though Durkheim sees regulation as comforting and supportive, there is a line between moral (and physical) authority being an anchor in a chaotic storm and it being a source of domination and oppression. This line, as we shall see, has obscured the role inequality, stratification, and oppression play in suicidality. In short, regardless of the importance of Durkheim’s basic insights, they fall short of helping us understand (a) why a particular person dies by suicide and (b) the mechanisms through which external social forces get inside someone’s psyche generating pain and rendering them vulnerable to suicide.

In this next section, we map sociological advances in understanding suicide by focusing on the new *structural* and then *cultural/social psychological* approaches that have emerged over the last two or three decades. To be sure, Durkheim’s approach continues to loom large over sociology, with a recent review lamenting the sheer lack of new approaches to the sociology of suicide (Wray et al., 2011), and thus while we highlight all major scholarship and theoretical contributions as possible, the basic dearth in research programs or teams is a more general limitation of the sociology of suicide. Like Durkheim, these theoretical and methodological projects build on the idea that there are emergent, distinct properties that are not reducible to the individual and her perceptions or decision-making. Yet, they do not deny the importance of intra-personal factors, instead they seek to supplement them. Collectively, these advances have great significance for general theories of suicide and for suicide prevention.

### Structural Insights

One of the first big innovations to Durkheim’s macro theory was to incorporate advances from structural sociology – and namely insights from social network theories – to elaborate how social integration and regulation matter to suicide. Social structure is a notably elusive concept, but it usually refers to sets of stable social arrangements that evince certain properties regardless of the specific incumbents. Social structures deeply shape individual life chances (Fourcade and Healy, 2013) by sorting us into particular opportunities, experiences, subcultures, social roles and obligations. They can be both easy to measure, as in the neighborhoods we live in or the schools we attend, or complex and intangible. Network theories facilitate the identification of local social structures that are salient to the individual and more closely capture the reality of the social world that surrounds them in their daily lives (Perry et al., 2018).

One of the greatest advances in sociology of suicide is the social network elaboration of Durkheim’s theory. This approach allows for greater specificity of social structures and cross-fertilization with contemporary social theory. With Durkheim’s “societies” translated into the operation of different networks, solidarity comes from the presence (or absence) of strong, interlocking social relationships. The power of the external social world is preserved, while situating the individual more realistically in it. Another advantage of a network approach is that it avoids the overly optimistic view of personal ties as always protective. Indeed, a plethora of work within the social network perspective has long demonstrated that the presence of *negative* ties is potentially more powerful in affecting individual well-being than positiveties (Abrutyn and Mueller, 2014a; Perry et al., 2018).

**FIGURE 1:**
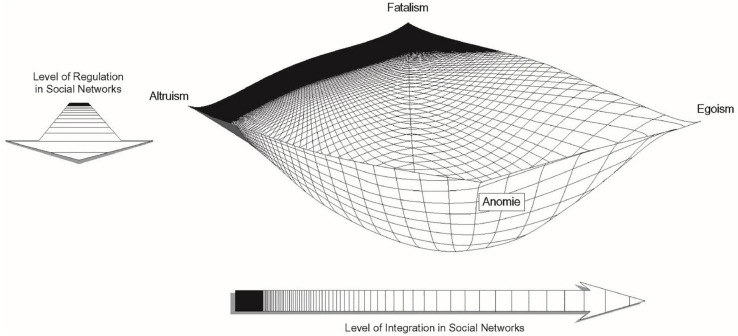
Networks and the Durkheimian theory of suicide.

Perhaps most importantly, a network approach highlights how integration and regulation coexist and in fact likely co-determine place-based vulnerability to suicide. An idea that is contrary to Durkheim’s four distinct “ideal-types” of suicide (egoistic, anomic, fatalistic, and altruistic). Instead, scholars advanced a curvilinear theoretical predictive plane with four dangerous poles matching Durkheim’s types (as seen in Figure 1). One dimension, running from left to right, represents integration. Another dimension, running from back to front, represents regulation. Both dimensions run from high to low, and their interaction generates the four types of suicide. When individuals live in social structures characterized by too little integration or regulation, the threads of the social safety net are too far apart to catch them when crises destabilize their equilibrium. Egoistic and anomic suicides are then theorized as “diseases of the infinite” because of the extreme gaps in the societal safety net that normally support individuals during times of individual or community crisis. Conversely, the social safety net closes up when social structures are overregulated or over-integrated. With no flexibility or give in the safety net, individuals who experience crises hit a wall that shatters rather than supports. It is in the center of the net, where ties are moderately integrated and regulated that individuals can be safely caught and restrained from their suicidal impulses (Pescosolido and Georgianna, 1989; Pescosolido, 1990, 1994).

A recent study illustrates the power of this structural approach. Using novel US data connecting the social profile of individuals to the social profile of the communities where they live, the study draws from social network theory’s principles of selective attachment or homophily (i.e., the tendency of individuals to connect with similar others, sometimes called “assortative relating” in psychology) and differential association (i.e., individuals sometimes come to behave more like those with whom they interact) (Pescosolido et al., 2020a). Specifically, researchers examined whether the presence of more “like” or “similar-others” would affect individual suicide risk and found that community “sameness” generally reduces individual risk of suicide. This multi-level examination of individuals’ embedded lives provides a glimpse into how “sameness” taps into structural ties, normative climates, and social diffusion processes. In fact, some of the most robust suicide research findings at the individual level are dramatically moderated by a consideration of their social environmental counterpart (Muller et al., 2020; Pescosolido et al., 2020a). Recent research further reaffirms the notion that current gaps in societal safety nets contribute to emotional distress and suicidality during the COVID-19 pandemic (Fitzpatrick et al., 2020).

Collectively, these findings suggested a critical and fundamental sociological insight into suicide: connectedness is protective *to a point.* Where there are too few others at risk (e.g., the employed in an upper middle-class community), socially supportive ties may be unavailable but when others share the same fate, the sense of individual failure transforms into structural failure (e.g., unemployed in a rust-belt community), potentially reducing the psychological harm of the experience. But when that sense of despair or fatalism engulfs the community as a whole, the ability to see *any* future can be restricted in isolated and historically stigmatized communities (Pescosolido et al., 2020a). These studies illustrate how essential it is to consider the roles of social structure and culture in social interaction, as a core feature of theories of suicide; to not do so contradicts basic contributions to contemporary population health research (Pescosolido, 1992; Hall and Lamont, 2009). It also illustrates that while Durkheim offered foundational insights into suicide, focusing overly on his specific hypotheses rather than the general theoretical propositions or attempting an artificially general theory of suicide, only weakens our capacity to understand how the external social world shapes suicide. And while structural insights into suicide represent major advances, contemporary sociological research raises two theoretical issues that cannot be ignored, and must be synthesized into, the understanding of suicide as a complex phenomenon – culture and contagion.

#### Exposure to Suicide

While Durkheim presented himself as a general theory of suicide, there are intricate aspects of social interaction that fall outside his purview but are related to how social structures and connectedness impact suicide. A second major line of sociological scholarship examines exposure to suicide through one’s social networks and communities and in so doing offers perhaps the clearest example of how social ties can produce harm (Abrutyn and Mueller, 2014a). Decades of research from a variety of methodological approaches and causal modeling strategies has confirmed that exposure to (1) media reports of suicides (Stack, 1987, 2005, 2009; Gould, 2001; Romer et al., 2006; Gould et al., 2014)– especially celebrity suicides – or (2) personal role models, like parents or friends (Abrutyn and Mueller, 2014a; Mueller and Abrutyn, 2015; Mueller et al., 2015a; Randall et al., 2015; Fletcher, 2017; Myfanwy et al., 2017), is associated with increased risk of suicidality. This line of research emerged from a series of studies by sociologist David Phillips (1974, 1979) that found that suicide rates among audiences exposed to media reports of suicides would spike temporarily. At the time, this was radical in that Durkheim famously denied the roles of micro-sociological processes related to social interaction, as well as diffusion or contagion in suicide. Phillips turned to a forgotten sociologist, Gabriel Tarde, to think theoretically through what he came to call suicide *suggestion*, a term derived from Tarde, who wrote about the diffusion of ideas and behaviors through social relationships (Abrutyn and Mueller, 2014b). Tarde was what would be called a social psychologist, but in the late 19th/early 20th century, his epistemology was too close to psychology, and thus Durkheim rejected it out of hand. Durkheim firmly committed to the idea that larger structural forces were *causal*, and thus, he is usually understood as rejecting the idea that suicide could “spread” or be “socially contagious.” And while research has repeatedly found, using more conservative methods than Phillips, an association between media exposure and increases in suicide rates, like Durkheim, these studies fall short in their ability to identify the primary mechanism or mechanisms that link the media exposure to the individual-level actions.

Nevertheless, a series of promising studies emerged following Phillips’ work, which focused on the consequences of being exposed to a personal role model’s suicidality (Tishler, 1981; Farberow et al., 1987; Niederkrotenthaler et al., 2012). With the growth of network analysis in the 21st century, suicide scholars in this burgeoning tradition began taking cues from network studies that found many social behaviors, like obesity and smoking, were socially “contagious,” net of individual factors (Christakis and Fowler, 2007, 2008). It became apparent that the structure of a person’s social network mattered, as longitudinal research found that adolescent exposure to friends of friends was associated with greater risks of suicidality (Baller and Richardson, 2009). Likewise, networks appear to have gendered effects, with girls being most at risk of suicidality when they have exceedingly small social networks or are immersed in exceedingly large ones (Bearman and Moody, 2004). Additionally, in a groundbreaking study, Baller and Richardson (2002) used spatial analysis to determine how crucial characteristics of place – like the degree of infrastructure – are to the clustering of suicides in places. They concluded that the structure of place and diffusion processes cannot be divorced from each other; once again illustrating the importance of theorizing and modeling the multiple levels of society within which human behavior is situated. Despite these advances, the question remained *why* and *how* suicide contagion worked.

While this is still an area in need of further exposition, in one unique study, researchers leveraged network data with pairs of adolescent friends to determine whether knowledge of a suicide attempt was necessary for suicide contagion to occur (Mueller and Abrutyn, 2015). The study found that youth who did not know their friend had attempted suicide were not at higher risk of suicidality over time, though if they did know they were. Additionally, exposure to a friends’ suicidal thoughts was not sufficient to increase risk of suicidal thoughts or behaviors 1 year later. These findings suggest the power of behavioral role modeling. How, why, and when social behaviors diffuse through social networks or contexts is an important and on-going area of inquiry within sociology of suicide specifically (Abrutyn et al., 2019) and social network science more generally (Kadushin, 2012).

#### Regulation, Culture, and Behavior

While these structural sociological theories described above offer multiple important advances for the sociology of suicide, they leave several unexplored social scientific questions – specifically, what mechanisms translate structure into meaningful social beliefs and practices that shape our attitudes and behaviors related to mental health and, ultimately, suicidality. Arguably, these gaps in the sociology of suicide can be addressed by drawing on insights from the broader theories in the sociology of culture and sociological social psychology. The incorporation of culture and sociological social psychology matters for several reasons. Eschewing explanations that motivate behavior by intra-personal perceptions, sociologists have generated substantial evidence that individual behavior is motivated – and justified – in reference to the web of social relationships and the broader structures and cultures in which these are embedded (Vaisey, 2009; Lizardo et al., 2016). We begin by reviewing theoretical advances that reconceptualize Durkheim’s regulation as a cultural force to better elaborate how culture shapes behavior and suicide.

Durkheim’s choice of regulation as a key causal force was rooted in the idea that collective ways of acting and thinking not only reinforced integration – that is, everyone is or is believed to be doing the same things, and thus share more than they differ – but that they were psychologically, emotionally, and socially healthy (Bearman, 1991). Although Durkheim could not imagine using cultural analysis, his conceptual ideas about regulation square quite neatly with contemporary cultural sociology. Groups of all sizes have cultures, and these cultures are shared – within reason – providing individual members with a sense of who they are, what they are supposed to feel, think, and do under various conditions, and what it means to belong to that group (Fine, 2010). Culture is activated every time members interact in real life or when one member anticipates or imagines interacting with another member; culture is also activated whenever we come into contact with externalized representations of it (Patterson, 2014), such as a Catholic individual seeing a crucifix. Members watch each other and sanction each other (see networks) to regulate each other’s behavior. However, culture also is internalized in our conceptions of the generalized other: people do not just act because they do not want to be sanctioned by others, but rather are motivated to act by the cultural schema, scripts, and frames they are exposed to and internalize and come to take for granted as normative (D’Andrade, 1984; Vaisey, 2009; Lizardo et al., 2016).

This set of insights is fundamental to explaining social behavior of all kinds but has largely been neglected in suicidology, even as structural and psychological accounts of suicide have been criticized since the 1960s for ignoring the role cultural meanings play in understanding and explaining variations in suicidality across time, space, and groups/classes of people (Douglas, 1967; Farberow, 1975; Baechler, 1979). And though it may be tempting to dismiss cultural regulation as a causal mechanism, research on other types of behavior shows culture not only shapes us; it regulates us morally – that is, it may proscribe *or* prescribe a behavior as a *normative* option under a shared set of conditions.

This is imperative for suicidology for two reasons. First, the last two decades have seen theories attempting to explain how suicide ideation is transformed into action expand dramatically (Joiner, 2005; Klonsky and May, 2015; O’Connor and Kirtley, 2018). Second, these theories largely neglect the simple fact that suicide is a *social act* and therefore is replete with cultural meanings (Boldt, 1988; Kral, 1994) that attempters symbolically externalize to their intended and unintended audiences, who make sense of the suicide via meanings they too have internalized. Put in the language of many current psychological theories, cultural sociology argues that suicide is not just about acquiring the proper cognitive and practical capacities to attempt, but also the *normative* capacity, or the belief that suicide is a viable and socially acceptable option for expressing outwardly something felt internally (Canetto, 1993; Kral, 1994; Abrutyn et al., 2019).

Recent decades have seen a growing body of historical, anthropological, and sociological evidence supporting the argument that culture matters to suicide. Research clearly demonstrates that societies and/or subgroups within those societies carry different beliefs about suicide across time and space (Barbagli, 2015) and death more generally (Long, 2004). These beliefs, ultimately, contribute to notions of when suicide is justified (Canetto, 1993; Hecht, 2013), if ever, and, therefore, erect prohibitions for entire classes of people or may make suicide a *normative* option (Niezen, 2009; Fei, 2010; Kitanaka, 2012; Abrutyn, 2017). This argument extends beyond whole cultures and applies to subpopulations and their subcultures. For instance, research has shown that how Americans interpret the suicides of men and women is through very different “cultural scripts” (Canetto, 1993, 1997), which has consequences for how their performed suicidality may be expressed and received by both the attempter and her intended (and unintended) audience (Hjelmeland et al., 2002), and, for which type of person might be at risk of suicide under certain conditions (Canetto, 2015). Other research has found distinctive beliefs and, subsequently, suicidal practices among young Latinas in the United States (Gulbas et al., 2015), in rapidly growing urban spaces in southern India (Chua, 2014), and some Indigenous communities in the United States (Tower, 1989) and Canada (Kral, 2012).

A second body of research underscoring the role of culture in suicide comes from a clinical psychology of bereavement. In short, Robert Neimeyer and his many collaborators have demonstrated that sudden deaths, like suicides, are shocking and compel individuals to make sense of them, to sift through available meanings as part of the bereavement process (Gillies and Neimeyer, 2006; Neimeyer et al., 2014). Though not a sociologist, Neimeyer and colleagues repeatedly find that meaning-making and bereavement always occur within the confines of a collective, as they build a coherent sense of why the death happened through each member’s individual meanings and more general societal ones (Neimeyer et al., 2006; Currier et al., 2015). In the event that collective meaning-making fails or that unhealthy meanings are arrived at, bereavement can become prolonged, thereby placing the individual at a significantly higher risk of emotional distress and suicidality.

A similar set of studies examine how structure and culture interact together, marrying Durkheimian insights to some of the more innovative cultural studies. For instance, research in Indigenous communities has made important connections between the social, cultural, and geographic circumscription that delimits social networks within some indigenous communities to the intergenerational negative affect experienced and passed on due to discrimination and prejudice (Kral, 2012; Stevenson, 2014). In one community, for instance, youth associated suicide with *belongingness*; that is, to die by suicide was to express one’s commitment to the group’s expectations and its members (Niezen, 2009). In rural China, Fei (2010) also identified linkages between structure and culture: where traditional patriarchal families tightly constrained women’s ability to express grievances, suicide had become means of expressing grievance, justice, and anger. Finally, in a recent publication, sociologists Muller et al. (2020) leveraged extremely unique longitudinal data linked to death records to examine how male adolescents’ desired occupations translated into risk of suicide by mid-life when those occupations became unavailable due to economic declines in those occupations. The structural changes in the labor market interacted with cultural ideals for work and success, such that when worked declined, men who expected a reliable working-class job were more likely to die by suicide (and also drug overdose) than their peers. This study suggests that it is not simply occupational or education attainment that generates risk of suicide, and not simply economic societal changes; but rather, the macro-societal translates into distress through an individual’s cultural values, identities, and expectations.

A third set of studies revolved around an in-depth ethnographic case study of a community called “Poplar Grove,” a white, affluent, homogeneous community with an intense high-pressure culture revealed that youth and parents alike had developed suicide explanation that had expanded for whom suicide was an option (Abrutyn et al., 2019). Youth believed other youth used suicide to escape the intense pressure and that the misery induced by the pressure caused suicide (Mueller, 2017; Abrutyn et al., 2019). Though more research is necessary on this (for some promising studies, linking attitudes to suicide see Gould et al., 2014; Phillips and Luth, 2018), this study suggests that identification with perceived and socially legitimated motives for suicide may increase youth’s vulnerability to suicide and may be one explanation for why suicide clusters form and persist (Mueller and Abrutyn, 2016; Mueller, 2017; Abrutyn et al., 2019). Further teasing out the mechanisms that translate external social environmental factors into internal psychological pain is a crucial project for the sociology of suicide. One strategy is to integrate principles drawn from sociological social psychology; a project the sociology of suicide has recently begun and which we turn to next.

### The Necessary Role of Social Psychology

Although Durkheim was not and could not be a social psychologist, contemporary sociological social psychology offers key mechanisms for understanding and explaining suicide within the context of structural and cultural contexts. Durkheim recognized in *Suicide* that individual’s membership in a specific group or category of people made them more or less vulnerable based on that collective’s integrative and regulative characteristics. Contemporary accounts have extended these insights, linking them to individual feelings or beliefs about who we are and what we are supposed to be doing. However, it is the mechanism linking us to the group, or what sociological social psychologists call *identities* and the *emotional* attachment we have to our identities and to the group that help us make sense of why structures and cultures may be harmful or protective.

The basic premise of a social psychological theory of suicide, then, rests on four key aspects of identity and emotion (Abrutyn and Mueller, 2016). First, persons whose identity is structurally and culturally embedded in a relationship, group, or broader social system will feel higher levels of commitment to the identity. Commitment depends on both intensive (intimate and affectual) and extensive (dense and numerous) social ties that evoke the identity (Stryker, 2008). Second, where commitment to an identity is high, the person will also be affectually attached to the bond itself (Lawler, 2002). Third, the more committed an individual is to an identity and attached to a bond, the more influence other members have on the feelings, thoughts, and actions of the individual. Fourth, where fewer alternative identities and bonds exist, subjectively and/or objectively, cultural regulation will be at its most powerful as continued commitment and attachment are more desirable than exclusion and isolation (Goffman, 1961). Below, then, we examine a little more closely what identities are and why emotions, especially social emotions, can help explain suicidality.

#### Identity

Identities are internalized meanings that cluster around how an individual understands themselves, as a social object, in relationship to a real person (one’s child), a group (e.g., family or congregation), a social class (e.g., race, sex, and occupation), or an abstract collective (e.g., American), which are embedded in social structure (Stryker, 1980; Burke and Stets, 2009; Hogg, 2018) and culture (Abrutyn, 2014). In turn, like the example of Catholic objects imbued with collective emotions and public meaning, our identities are objects inseparable from the collectives they are anchored, which makes them as emotionally charged as the external objects themselves. They matter to us because the relationships that allow them to exist matter. And, like any object that takes on meaning in interaction, relationships are where people acquire these identities as they learn about who they are, the expectations that others have of them and that they have others, what rewards, performances, and influence they can expect to have, and so forth. Identity matters, then to suicide and mental health, because it is one prominent pathway through which the external social world comes to matter to perceptions of self. Our identity renders painful the possibility of exclusion, rejection, and isolation from cherished social groups, not simply because we feel lonely, but because a part of our self can be damaged or lost through these social experiences. And, when we assign blame to our self for the rejection by a group (etc.), emotions signaling we are “bad” or “worthless” may snowball into psychache (Shneidman, 1993) and negative emotion feedback loops (Scheff, 1988).

Returning, then, to the study of Poplar Grove, youth in this community did indeed internalized a very clear, rigid, coherent sense of what was expected of the “typical” Poplar Grove youth (Mueller and Abrutyn, 2016; Mueller, 2017). The small nature of the community delimited the variation in how this identity could be performed, and thus made even the counterfactual cases we spoke with painfully aware of expectations. And, because the school took on an outsized role in community life, this identity was trans-situational, defining nearly all of the relationships inside and outside of school. This had three key consequences for the suicide problem in Poplar Grove. First, youth had also internalized the cultural script of pressure leads to emotional distress, which can lead to suicide being a normative option for expressing one’s identity and extinguishing the pain. Second, the community had set most of the kids up to fail, as only one kid could be captain of the football team, lead actor in the big school play, or most popular kid. Anything short of five AP classes per semester and straight A’s was viewed as a failure by youth, making falling short of expectations the norm and not the exception. Third, fear of failure, imagining or anticipating failure, and actual failure all lead to the same thing: shame (Abrutyn and Mueller, 2016; Mueller and Abrutyn, 2016). Shame is a painful social emotion signaling that the person has not only not met expectations but are actually a “bad” person because of doing so; it is social in that they believe, whether true or not, that others judge them as deficient. Identities are intimately implicated in this process, as not meeting expectations generate negative affect that compels us to meet them (Burke and Stets, 2009), but because of the second consequence described above, failing was perceived as a chronic, normal state of adolescence. And thus, we must consider, in a bit more detail, the role of emotions in suicidology.

#### Emotions

Generally speaking, suicidology has focused on cognitive appraisals of emotions (Cavanaugh et al., 2003), as opposed to the affect themselves, which is very often shaped by the cultural world around us. Emotions are both the “glue” of social relationships and can signal our successful integration (Lawler, 2002) or fulfillment of obligations or expectations, or our failure to do so; and, as such, are a fundamental element of how Durkheim’s regulation becomes internalized into psychological well-being or pain (Scheff, 1997; Lewis, 2003). Thus, emotions create and sustain attachments to others and our own commitment to the identity associated with the attachment. In turn, this level of integration engenders greater regulation as we are more likely to adopt the feelings, thoughts, and actions of those we are most affectually attached (Lawler et al., 2009). On the other hand, emotions, particularly negative social emotions like embarrassment, guilt, or shame, are the signals that this connection is in danger, dissolving, or lost (Abrutyn and Mueller, 2014c). The link between identity and culture points, then, to two key insights drawn from scholarship on emotions and behavior. First, when we are not performing our identities as others expect or as we expect, we feel negative social emotions like embarrassment, guilt, and shame (Lewis, 1971; Scheff, 1997). What makes us feel bad about ourselves, or creates the cognitive appraisals like worthlessness or hopelessness, is very much a product of the cultural milieu that provides us with expectations about who we are and why we are supposed to do. Second, depending on the structural and cultural context, these social emotions may endure over time, making it increasingly difficult to live up to expectations and overwhelming our ordinary cognitive and behavioral functions, leading us to draw from existing cultural options for dealing with those emotions.

In particular, shame or the social emotions that that the self is viewed as being corrupt, polluted, deficient, and contemptuous by others – objectively or not – plays a key role (Abrutyn and Mueller, 2014c). Research has demonstrated the role shame plays in a range of negative behaviors, such as domestic violence (Lansky, 1987), eating disorders (Scheff, 1989), and criminality (Braithwaite, 1989). It also has some anecdotal links to suicide (Mokros, 1995; Lester, 1997; Kalafat and Lester, 2000). The shame pathway, then, can be tied directly to our discussion of social psychology, identity, and expectations: failing to meet expectations can trigger shame. In part, this may be due to the publicly shared cultural meanings. For instance, research in cultures or subcultures with strong traditional male norms evince far more “honor” suicides as failure to meet masculine expectations are closely tied to suicide as a way of restoring honor (Adinkrah, 2012; Cleary, 2012). Sudden loss of status, in most cases, is followed by intense shame and the need to process the shame. Shame also plays a role for those in subordinate positions, whose identities are wrapped up in being powerless. In some traditionally patriarchal societies, like rural China (Fei, 2010), there may be no other culturally available recourse to processing their shame besides suicide. Indeed, as Zhang’s (2010; Zhang et al., 2017) use of strain theory and innovative methods reveal, there are severe structural constraints on access to many legitimate means to reducing anxiety and stress. Youth, too, are in a relatively powerless position coupled with being at a disadvantaged cognitive and emotional developmental state that precludes being able to see far into the future. Shame can be experienced so acutely for these kids, the availability, accessibility, and applicability of a suicide script may be the only ingredient missing for leading to suicide vis-à-vis drug or alcohol abuse. Thus, social emotions are a powerful vehicle, particularly when rooted in salient social identities in valued social environments, through which the external social world is translated to internal psychological pain.

## Discussion

Sociological theories of suicide, inspired by Durkheim’s original work, help explain how the external social world matters to individual well-being and psychache, thereby revealing the social roots of suicide. The external social world is complex and multi-layered and can be characterized by network structures and shared cultures which in turn impact individual group members through their social identities and social emotions. That the external social environment matters to human development across the life course, including to physical and mental health, and even suicide, is not necessarily new. However, as rates of suicide have climbed in the United States and around the world, the importance of understanding the social environment’s roles in suicide and suicide prevention has become more prominent and even urgent (Wyman, 2014). Sociology, with our long tradition of specifying how society conditions human lives, is well situated to answer this call, while also building bridges into other disciplines.

### Implications for Psychological Theories of Suicide

Many psychological theories of suicide acknowledge social and environmental factors, facilitating the incorporation of sociological insights to suicide. For example, belongingness is critical to Joiner’s (2005) interpersonal theory (IPT) of suicide, and connectedness is a key component of Klonsky and May’s (2015) three-step theory of Suicide (3ST). There are two primary ways that sociological insights should, we argue, be incorporated into major psychological theories of suicide. First, while psychological theories of suicide recognize that the external social world matters, they generally distill the social world down to an individual’s perception of it (e.g., belongingness and connectedness). Sociological research suggests this is insufficient and that using strategies to measure the external social world independent of a person’s perception or experience is important. This could be as simple as using egocentric network methodology (Perry et al., 2018) to better measure the culture and structure of a person’s proximate social environments (Perry and Pescosolido, 2015; Perry et al., 2016). This approach would involve having focal research respondents report their multiplex network ties (often friends, family, etc.) using name generators and characterizing them through theory-informed name interpreters. An ideal research design then involves interviewing some of the nominated network ties, so that data does not rely solely on the focal respondents’ perception.

Second, structural-cultural insights into suicide reveal that cultural scripts for suicide that prevail in people’s salient social groups may impact their capacity for suicide (Canetto, 1993; Abrutyn et al., 2019; Mueller, 2017). While the notion of individuals’ capacity for suicide already exists (Joiner, 2005), recognizing that *normative* capacity – or how a group’s beliefs about why people die by suicide, who is expected to be vulnerable to suicide, as well as when, where, and how people suicide – contributes to making suicide an accessible and applicable option for an individual. Recognizing – and measuring – this may be a useful pathway for future research to examine; particularly given research linking explicit and implicit beliefs about suicide to suicide attempts and even death (Gould et al., 2004; Nock et al., 2010; Phillips and Luth, 2018).

### Implications for Suicide Prevention

Recognizing the importance of the social environment is also critical to strategies for suicide prevention. Some current suicide prevention strategies recognize the potential of broader, upstream environmental interventions, such as the Centers for Disease Control and Prevention’s (n.d.) emphasis on social connectedness in communities. The focus on building connectedness has also been leveraged to great effect in schools. Specifically, building trust between youth and adults in schools is associated with lower rates of suicidality among students (Wyman et al., 2010, 2019). Similarly, there are suicide prevention interventions that address cultural biases, like mental health stigma, in communities or schools (Wasserman et al., 2015; Pescosolido et al., 2020b). These interventions raise mental health awareness and normalize discussing mental health, which may foster help-seeking and diminish suicidality in the entire community. Additionally, suicide prevention strategies in healthcare – specifically so-called “Zero Suicide” approaches – promote changes in the social environment within healthcare organizations to improve medicine’s ability to prevent suicide (Labouliere et al., 2018). Specifically, a major component in the Zero Suicide model is generating system wide *cultural* change that renders suicide prevention a core organizational goal of any medical setting (Labouliere et al., 2018). Finally, recent research suggests that interventions into economic safety nets are associated with suicide rates; specifically, increases in the minimum wage are associated with meaningful decreases in suicide mortality (Gertner et al., 2019), perhaps especially when unemployment is high (Kaufman et al., 2020). This suggests that macro-level economic policies, untheorized as suicide prevention, may actually be powerful tools for just that.

While collectively these interventions show promise, limitations remain. For example, in terms of culture, much of these interventions focus narrowly on mental health stigma, despite substantial research that demonstrates a plethora of cultural beliefs that can promote vulnerability to suicide and its precursors. This may be particularly harmful when connectedness is leveraged in schools. Schools that house harmful youth cultures may find that intensifying connectedness, even when combined with positive mental health messaging, may, at worst, amplify their harmful culture or, at best, find that the unaddressed harmful culture undermines any positive cultural interventions (Mueller and Abrutyn, 2016). Similarly, with regard to organizational interventions like Zero Suicide, it is potentially not enough to encourage an organization to value suicide prevention and mental health; it is likely necessary to broaden the scope of research and understand the external pressures, obligations, or cultural directives the organization faces and examine how mental health and suicide prevention complements or competes with those other organizational directives. This critique is motivated by previous sociological research that shows that understanding how organizations balance competing goals is crucial to effective prevention (Perrow, 1999; Vaughan, 1999). Unfortunately, when organizations face external pressures (e.g., resource scarcity), public health safety is often deprioritized in favor of more dominant goals (see Vaughan, 1996).

### Future Directions

This last point highlights a broader limitation in suicidology that in turn points to a crucial future direction for research. Zero Suicide approaches are one of the only explicitly organizational approaches to understanding suicide or suicide prevention. In general, though we acknowledge the role of several key organizations [schools (Erbacher et al., 2014) and healthcare (Gordon et al., 2020)] in suicidology, we have largely neglected to theorize or examine empirically the role of organizations in suicide risk and prevention. This is a major limitation since suicide prevention largely takes place within formal organizations, and several formal organizations are implicated in suicide risk [e.g., occupations (Skipper and Williams, 2012), military (Bryan et al., 2012), and schools (Wyman et al., 2019)]. It is also a missed opportunity to leverage organizational science to improve suicide prevention. Within organizational science there are substantial literatures that have identified how to build safety systems to prevent hard to predict tragedies (Perrow, 1999; Vaughan, 1999), like suicide. An organizational approach to suicide prevention has other advantages, as it can help identify existing unused safety systems in organizations that could be leveraged for suicide prevention. For example, schools generally have existing multi-tiered systems of support – often for academic interventions (Eagle et al., 2015) or violence prevention (Payne and Elliott, 2011) – that potentially could be leveraged efficiently and effectively for suicide prevention (Harms, 2019).

There is a second critical future direction and current substantial limitation that warrants discussion. To date, theories of suicide largely neglect how structural inequality, colonization, and intersecting systems of oppression, privilege, and power shape vulnerability to suicide. Though there have been some exiting new efforts to theorize how structural inequality and intersectionality matter to suicidology (Brooks et al., 2020; Opara et al., 2020; Standley, 2020), much more work is needed. Based on broader research within the sociology of mental health – which does take up this issue – the patterns are likely to be complex and again not distillable to individual experiences with discrimination or prejudice (McLeod, 2015; Williams et al., 2019; Laster Pirtle, 2020). Prior research on mental health and inequality demonstrates that external social structures condition mental health above and beyond individual experiences (Sewell et al., 2016; Huyser et al., 2018; Williams, 2018). While it’s beyond the scope of this review to propose a new theory of inequality, power, and suicide, we can point scholars to useful theories of inequality in mental and physical health to aid them as we collectively take up this critical agenda (Phelan et al., 2010; Ridgeway, 2011; Carbado et al., 2013; Westbrook and Schilt, 2014; Phelan and Link, 2015; Sewell, 2016; Spencer and Grace, 2016). Additionally, understanding inequality will likely have real consequences for suicide prevention. For example, though upstream suicide prevention strategies are showing great promise in schools (Wyman et al., 2010; Wasserman et al., 2015), many schools struggle to sustain even evidence-based strategies over the long-run (Singer et al., 2019). This may be in part because many schools, particularly those that serve disadvantaged youth, experience intense resource scarcity (Leachman et al., 2017). Thus, considering the complex ways that inequality shapes suicide and suicide prevention is necessary to a robust, comprehensive theory of suicide.

## Conclusion

Sociology is best known for our Durkheimian insight into why people die by suicide – namely, that lacking meaningful social relationships that support us during difficult times and celebrate us when times are good is extremely harmful to individual well-being. However, a review of the full body of sociological scholarship, and especially the empirical and theoretical advances of the past 10 years, reveal the social roots of suicide. Incorporating sociological insights into how the external social environment can matter to suicide and suicide prevention may help us better understand the complexity of suicide and determine how to effectively intervene.

## Author Contributions

All authors contributed to writing portions of this manuscript and reviewing and editing it in its entirety. AM conceptualized the structure of the review.

## Conflict of Interest

The authors declare that the research was conducted in the absence of any commercial or financial relationships that could be construed as a potential conflict of interest.
